# Emerging Insights for Translational Pharmacokinetic and Pharmacokinetic-Pharmacodynamic Studies: Towards Prediction of Nose-to-Brain Transport in Humans

**DOI:** 10.1208/s12248-015-9724-x

**Published:** 2015-02-19

**Authors:** Mitchel J. R. Ruigrok, Elizabeth C. M. de Lange

**Affiliations:** Division of Pharmacology, Leiden Academic Centre of Drug Research, Leiden University, Gorlaeus Laboratories, Einsteinweg 55, 2333 CC Leiden, The Netherlands

**Keywords:** advanced mathematical modeling, blood–brain barrier, central nervous system, intranasal drug administration, nose-to-brain transport, translation

## Abstract

To investigate the potential added value of intranasal drug administration, preclinical studies to date have typically used the area under the curve (AUC) in brain tissue or cerebrospinal fluid (CSF) compared to plasma following intranasal and intravenous administration to calculate measures of extent like drug targeting efficiencies (%DTE) and nose-to-brain transport percentages (%DTP). However, CSF does not necessarily provide direct information on the target site concentrations, while total brain concentrations are not specific to that end either as non-specific binding is not explicitly considered. Moreover, to predict nose-to-brain transport in humans, the use of descriptive analysis of preclinical data does not suffice. Therefore, nose-to-brain research should be performed translationally and focus on preclinical studies to obtain *specific* information on absorption from the nose, and distinguish between the different transport routes to the brain (absorption directly from the nose to the brain, absorption from the nose into the systemic circulation, and distribution between the systemic circulation and the brain), in terms of *extent as well as rate*. This can be accomplished by the use of unbound concentrations obtained from plasma and brain, with subsequent advanced mathematical modeling. To that end, brain extracellular fluid (ECF) is a preferred sampling site as it represents most closely the site of action for many targets. Furthermore, differences in nose characteristics between preclinical species and humans should be considered. Finally, pharmacodynamic measurements that can be obtained in both animals and humans should be included to further improve the prediction of the pharmacokinetic–pharmacodynamic relationship of intranasally administered CNS drugs in humans.

## INTRODUCTION

Over the past few decades, an increasing effort has been put into research focusing on the central nervous system (CNS) and its disease conditions (1). This allowed the identification of new drug targets due to an improved understanding of CNS disease etiologies and pathologies. Consequently, scientists were provided with opportunities to develop novel drugs for CNS diseases. Despite this progress in neurosciences, the development of CNS-active drugs remains highly challenging as shown by relatively high attrition rates of drugs during clinical trials (2). Numerous candidate drugs for CNS diseases were efficacious during *in vitro* and preclinical *in vivo* studies. However, many of these drugs did not show efficacy when administered in humans. One important reason for this may be the lack of having the drug at the right time, at the right concentration, and at the right place (3).

The presence of the blood–brain barriers has typically been seen as an important reason for these problems and the intranasal (IN) route of administration has been implicated to circumvent these barriers, as direct absorption from the nose to the brain might exist (4). As human brain sampling is highly restricted, animal data should mainly provide insight into possible brain distribution enhancement via the IN route.

This review aims to provide insight in advanced experimental and mathematical modeling approaches using preclinical data, and proposed steps to be taken for translation between conditions and ultimately to species translatability for nose-to-brain transport in humans. To that end, the impact of the blood–brain barriers on drug distribution into the CNS is shortly discussed, followed by a summary on the knowledge of the nasal anatomy, histology, and physiology and their species differences; direct nose-to-brain drug transport mechanisms; evidence for direct nose-to-brain drug and drug delivery systems transport in animals; and evidence for direct nose-to-brain drug transport in humans. Then examples follow on the design of a translational preclinical pharmacokinetic–pharmacodynamic (PK-PD) study on remoxipride following intravenous (IV) and IN administration, and the successful PK-PD translation of IV administered remoxipride from rats to humans. All together, this information finally feeds into considerations and suggestions for future studies on translation of preclinical nose-to-brain PK and PK-PD data to the human situation.

## INTRANASAL ADMINISTRATION TO CIRCUMVENT THE IMPACT OF THE BLOOD–BRAIN BARRIERS ON DRUG DISTRIBUTION INTO THE CNS

Various drugs do not adequately reach CNS target sites due to the blood–brain barrier (BBB), the blood cerebrospinal fluid barrier (BCSFB), and the arachnoid barrier (5). These barriers not only protect the CNS from invading pathogens and various toxic substances but also provide an interface for blood–CNS exchange (6). The BBB is located in the endothelium of brain capillaries. The combined surface area of these brain capillaries makes it by far the largest blood–CNS interface. Therefore, most CNS-active drugs tend to enter the brain mainly by passing through the BBB.

Drug transport via the BBB can be limited in two ways. For hydrophilic drugs that cannot traverse cell membranes easily, paracellular transport across the BBB is highly restricted and only possible for the smaller sized ones, as tight junctions create a firm connection between adjacent endothelial cells. For the more lipophilic drugs that can pass cell membranes readily, transcellular passage of the BBB may be counteracted by the action of efflux transporter proteins, such as P-glycoprotein (Pgp) and multidrug resistance-related proteins (MRPs) that are present on the cell membranes of the brain capillary endothelial cells. Not all transporter proteins counteract drug transport across the BBB; some influx transporters actually aid the access of drugs to the brain. Thus, the BBB can play an important role in drug distribution into the CNS and therewith also in CNS target site distribution of drugs.

Knowledge of the BBB and its mostly limiting effect on CNS drug distribution has guided researchers to investigate and to develop novel drug delivery techniques which are capable of circumventing this barrier. Methods to bypass the BBB include opening of the tight junctions between endothelial cells to enhance the transport of hydrophilic drugs though paracellular diffusion (6). However, opening tight junctions also makes the brain more vulnerable to the entry of unwanted organisms and substances. The BBB can also be circumvented by using intracerebral implants and intraventricular infusions (7). Both of these drug delivery methods are very invasive and are usually only considered when no other methods are possible. So, there is a need for safer, easier, and less invasive brain drug delivery techniques which bypass the BBB.

Researchers answered to this need by exploring IN drug administration as a method to enhance the delivery of drugs into the brain while bypassing the BBB (8). This “direct nose-to-brain” transport is anticipated to offer several advantages in comparison to other drug delivery techniques. Firstly, IN delivery avoids the first-pass effect which improves the bioavailability of a drug. Secondly, there is a potential for direct nose-to-brain delivery as drugs could bypass the BBB. Thirdly, absorption of drugs by the nasal mucosa could produce a fast onset of therapeutic effects. Lastly, IN delivery is minimally invasive which may promote medication adherence in patients. Thus, direct nose-to-brain delivery could be a promising drug administration technique for patients who suffer from CNS diseases.

Many animal studies have shown that drugs administered via the IN route can enter the brain, although a relatively modest percentage of those studies present quantitative PK data to confirm the extent of direct nose-to-brain transport (9,10). Moreover, these studies were performed with animals, mostly rats, which have significantly different nasal features when compared to humans. This restricts the predictive value of preclinical animal models, though it is reasonable to assume that high direct nose-to-brain transport in humans is unlikely to occur if very low transport is observed in animals. It is anticipated that proper understanding of and progress in application of predictive approaches for PK and PK-PD data to be translated from animals and humans will lead to an improved predictive value. Ultimately, this will aid the translation of nose-to-brain transport from animals to humans. Therefore, first insight into nasal characteristics is essential.

## NASAL ANATOMY, HISTOLOGY, AND PHYSIOLOGY AND SPECIES DIFFERENCES

### Nasal Anatomy

**Fig. 1:**
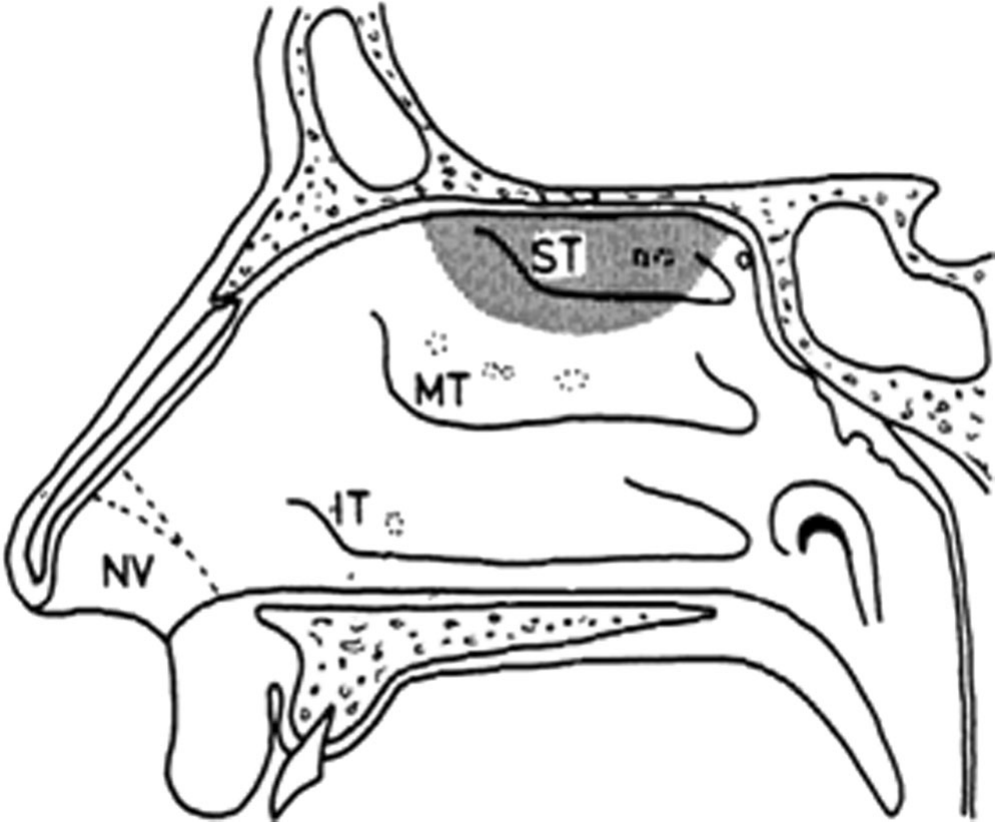
General anatomical features of the lateral wall of the human nasal cavity. *NV* Nasal vestibule, *IT* inferior turbinate, *MT* middle turbinate, *ST* superior turbinate

The human nasal cavity is a structure which connects the nostrils to the nasopharynx and is split in a longitudinal manner by the nasal septum (see Fig. 1) (11,12). In humans, the nasal cavity has an approximate length of 12–14 cm and a height of 5 cm. Furthermore, the nasal cavity has a surface area of 160 cm^2^. This surprisingly large surface area is partly created by the nasal turbinates. Nasal turbinates are horizontal bony structures shaped like scallop seashells. Each nasal cavity contains three turbinates (inferior, middle, and superior) which are present on the lateral wall. Additionally, the nasal cavity contains two functional regions which are concerned with (a) the conditioning and filtration of inhaled air before it enters the lungs (respiratory region) and (b) the sense of smell (olfactory region).

Another unique feature of the nasal cavity is its rich vasculature which aids the conditioning of inhaled air. Both the nasal septum and the lateral wall of the nasal cavity receive blood from the anterior ethmoidal, posterior ethmoidal, and sphenopalatine arteries (11). The nasal septum also obtains blood from branches of the superior labial artery and of the greater palatine artery. Additionally, the nasal vestibule gains blood from the lateral nasal artery. Finally, blood is drained into the ophthalmic vein, the cavernous sinus, and the pterygoid plexus.

Nerves in the nasal cavity are responsible for the transfer of chemosensory (chemicals and odors), nociceptive (pain), thermoceptive (temperature), and mechanoceptive (touch) information to the CNS. Firstly, olfactory sensory neurons (OSNs) are located in the olfactory region and their dendrites converge into the olfactory nerve which travels through the cribriform plate and ends up in the olfactory bulb. OSNs are bipolar neurons as they have only one axon and one dendrite (9). Their axons are located in the nasal epithelia where they extend surprisingly deep into the mucous layer. This feature benefits olfaction as it makes OSN receptors more accessible to odorants.

Furthermore, the respiratory and olfactory region are innervated by branches of the trigeminal nerve (i.e., the ophthalmic nerve, the maxillary nerve, and the mandibular nerve) which terminates in the trigeminal nuclei of the brainstem (9,13). However, the mandibular nerve does not innervate the nasal epithelium. Hence, innervation of the olfactory region and the respiratory region is only provided by the ophthalmic nerve and the maxillary nerve, respectively. Finally, free nerve endings of the ophthalmic nerve and the maxillary nerve extend into the surface epithelium, but they do not reach further than the level of tight junctions (14). Here, they transmit chemosensory information from the nasal cavity to the brain.

### Nasal Histology

Four types of epithelium can be found in the nasal cavity: squamous, transitional, respiratory, and olfactory (15). These types of epithelium line the surface of distinct regions in the nasal cavity. Firstly, squamous epithelium covers the nasal vestibule and it extends to the anterior side of the inferior nasal turbinate. Secondly, a narrow lining of transitional epithelium connects squamous epithelium with respiratory epithelium and respiratory epithelium with olfactory epithelium. Thirdly, respiratory epithelium lines the main chamber of the nasal cavity and the nasopharynx. Lastly, olfactory epithelium is present on the cranial side of the nasal cavity.

Squamous epithelium is composed of lightly keratinized stratified squamous cells and basal cells. Like the skin, squamous epithelium is thought to protect underlying tissue from toxic substances. Transitional epithelium consists of non-ciliated microvilli-covered cells and basal cells. Luminal non-ciliated transitional epithelium contains an abundance of smooth endoplasmic reticulum which might play a role in the metabolism of inhaled xenobiotics (15). Respiratory epithelium is mainly comprised of ciliated pseudostratified cells, although it also consists of mucous, non-ciliated, columnar, cuboidal, brush, and basal cells. Several xenobiotic metabolizing enzymes (e.g., carboxylesterase, aldehyde dehydrogenase, and cytochrome P-450) have been identified in respiratory epithelium (15). Lastly, olfactory epithelium is composed of three cell types: olfactory sensory neurons, sustentacular cells, and basal cells. Upon neuronal cell loss, olfactory sensory neurons regenerate (neurogenesis) to preserve the ability of olfaction.

The respiratory and olfactory epithelia are the main absorption sites for direct nose-to-brain drug delivery. However, olfactory epithelium is the most relevant for direct nose-to-brain delivery as it provides a direct link between the nasal cavity and the CNS which bypasses the BBB (13).

### Nasal Physiology

The nasal cavity has several important functions. Aside from olfaction (i.e., the sense of smell), the nasal cavity is involved in conditioning inhaled air (12). Inhaled air is heated, humidified, and filtered before it enters the lungs, which limits bronchial heat loss and tissue damage. Arteriovenous anastomoses in the nasal cavity aid the exchange of heat from arterial blood to inhaled air. Furthermore, the small width of the nasal cavity facilitates close contact between the mucous layer and inhaled air. Consequently, the nasal cavity conditions room air from 23°C with a relative humidity of 40 to 32°C and a relative humidity of 98%.

In addition, inhaled air is filtered before it enters the lungs because clearance of harmful agents becomes significantly more difficult when they are deposited into bronchi and alveoli. Filtration of air occurs as harmful agents are deposited into mucus which is present on the surface epithelium of the nasal cavity. Mucus is produced by Goblet cells and it contains antimicrobial enzymes, immunoglobulins, and lactoferrins (16,17). Furthermore, mucus is constantly pushed towards the nasopharynx by motile cilia which are present on ciliated cells. Finally, the fate of mucus is either expectoration or swallowing. This whole process is called mucociliary clearance, and it results in a nasal clearance half-time of approximately 15 to 20 minutes for most non-mucoadhesive substances (18). Interestingly, olfactory epithelium lacks cilia mobility which results in reduced mucociliary clearance. On the other hand, respiratory epithelium contains ciliated cells, resulting in more prominent mucociliary clearance (13).

### Species Differences

For the predictive value of preclinical animal models for the human situation, it is important to realize that major differences exist in nasal features between conditions, such as species differences, especially between animals and humans (9,19,20). Rats are used most often as a preclinical animal model in direct-nose-to-brain drug delivery studies, although mice and rabbits are used as well. In Table I, it can be seen that the relative absorption area is much larger (8-fold) in rats than in humans. Humans have an average bodyweight, a nasal cavity volume (NCV), and a nasal cavity surface area (NCSA) of 70 kg, 25 and 160 cm^2^, respectively. This yields a relative surface area (NCSA/NCV) of 6.4 cm^−1^ in humans. Rats have an average bodyweight, NCV, and NCSA of 0.25 kg, 0.26 and 13.4 cm^2^, respectively. Therefore, it can be generally said that direct nose-to-brain delivery obtained from rats will overestimate direct nose-to-brain transport in humans if differences in the relative surface area are not adequately accounted for.

**Table I:** Anatomical and Histological Differences Between The Nasal Cavity of Mice, Rats, Rabbits, and Humans (9,11,12)

Parameter	Mice	Rats	Rabbits	Humans
Bodyweight (kg)	0.03	0.25	3	70
Nasal cavity volume (cm^3^)	0.03	0.26	6	25
Nasal cavity surface area (cm^2^)	2.89	13.4	61	160
Relative surface area (NCSA/NCV)(cm-1)	96.3	51.5	10.2	6.4
Olfactory epithelium (% of NCSA)	47	50	10	8
Olfactory epithelium (cm^3^)	1.37	6.75	6	12.5

The relative surface area of the nasal cavity in mice, rats, and rabbits is higher than in humans. Furthermore, mice and rats have a substantially higher relative amount of olfactory epithelium (% of NCSA) than humans (factor 15 and 8, respectively) (9,11,12)

## NOSE-TO-BRAIN DRUG TRANSPORT MECHANISMS

IN administered drugs can reach the CNS via three transport mechanisms (13). Firstly, drugs can enter the CNS via the olfactory bulb by transport along the olfactory nerve. Secondly, drugs can enter the CNS by transport via the trigeminal nerve. These two drug transport mechanisms circumvent the BBB, resulting in direct nose-to-brain transport. Lastly, drugs can enter the systemic circulation by absorption through arteriovenous anastomoses. Thereafter, drugs in the systemic circulation can enter the brain by crossing the BBB. Collectively, these mechanisms can be used to deliver drugs from the nasal cavity into the CNS.

### Olfactory Nerve Direct Nose-to-Brain Transport

The olfactory nerve is likely to be the most important in direct nose-to-brain drug delivery, although more research is necessary in this field (18,21,22). OSNs in the olfactory region regenerate every 3–4 weeks which may result in a decreased barrier function due to a combination of factors. For instance, tight junction proteins, efflux transporters, and proteolytic enzymes might not be fully present or functional during the regeneration process, resulting in a “leaky barrier” (13,23). This decreased barrier function can be exploited to achieve direct nose-to-brain drug delivery. Intranasally administered drugs can reach the CNS via the olfactory pathway in two ways. Firstly, drugs can diffuse into the extracellular spaces of the olfactory nerve bundles. Subsequent transport to the olfactory bulb is likely to depend on a combination of bulk movement and propagation of action potentials along the olfactory nerves. Secondly, drugs can be transported via intracellular mechanisms such as passive diffusion, adsorptive endocytosis, and receptor-mediated endocytosis. However, extracellular transport along the olfactory nerves is faster (minutes to half an hour) than intracellular transport (hours to days) (13). Upon reaching the olfactory bulb and cerebrospinal fluid (CSF), drugs can access other regions of the CNS by bulk flow mechanisms and by mixing with brain extracellular fluid (ECF).

### Trigeminal Nerve Direct Nose-to-Brain Transport

Direct nose-to-brain drug delivery can also occur to a lesser extent via transport along the trigeminal nerve. Branches of the trigeminal nerve innervate both the respiratory and olfactory regions in the nasal cavity, as mentioned earlier. After passing through the respiratory and olfactory epithelium, drugs can move along the trigeminal nerve via intracellular or extracellular transport mechanisms where they can enter the brain through either the cribriform plate or the pons. Interestingly, the distance between the nasal epithelium and the brainstem (trigeminal pathway) in rats is estimated to be ∼20–30 mm, while the distance from the olfactory epithelium to the olfactory bulb (olfactory pathway) is estimated to be ∼4–5 mm. Once present in the brain, drugs can diffuse through the CSF and brain ECF in order to reach other CNS regions. Unfortunately, the degree of transport attributable to this route is not fully understood.

### Nose-to-Brain Transport via Initial Absorption in the Systemic Circulation Followed by BBB Transport

Lastly, various small molecules and biomacromolecules administered via the IN route have been shown to enter the CNS by initial absorption into the systemic circulation and subsequent transport through the BBB (24). Initial absorption into the blood is restricted by three barriers: the mucous layer, the epithelial membrane, and the junctional barrier (16). Initial diffusion of drugs into mucus has been shown to be highly dependent on lipophilicity (25). Next, drugs have to cross the nasal epithelium by either transcellular or paracellular transport. Lipophilic drugs cross this barrier via transcellular transport by partitioning into the lipid bilayers of cells. Additionally, transcellular transport can also occur by receptor-mediated or vesicular transport (26). Paracellular transport of hydrophilic molecules is highly limited by its weight as they have to passively diffuse through tight junctions. Generally, paracellular transport only occurs when drugs have a molecular weight <1000 Da (26). Afterwards, drugs which passed these barriers will enter the venous blood flow. In order to reach the CNS, drugs also have to pass the BBB, which has been addressed shortly in a previous section. In conclusion, drugs can also reach the CNS via initial absorption into the systemic circulation, followed by blood-brain transport.

### Physicochemical Properties of Drugs

Absorption of drugs via the nasal cavity into the systemic circulation is highly dependent on physicochemical properties of drugs (24). However, not much is known about statistically significant correlations between the efficiency of direct nose-to-brain transport and the physicochemical properties of IN administered drugs (10,27). Although many different drug types (small molecules, biomacromolecules, and specialized drug delivery systems) have been shown to enter the brain via direct nose-to-brain transport, both Kozlovskaya *et al*. (2014) and Lee *et al*. (2010) were unable to identify correlations between the extent of direct nose-to-brain transport and the molecular weight (MW), partition coefficient (LogP, which describes the partition of unionized compounds in two immiscible phases), and distribution coefficient (LogD, which describes the total partition of both ionized and unionized compounds in two immiscible phases, and as such is dependent on pH) (10,27). However, these results are in contrast with the findings obtained by Sakane *et al*. (1991) and Chou and Donnovan (1998) who observed a statistically significant correlation between lipophilicity of compounds and drug concentrations in the CSF after IN administration (28,29). On the basis of these studies performed so far, no conclusions can be drawn about the influence of physicochemical properties of drugs and nose-to-brain transport, and further investigations are needed to allow a more rational approach to drug design.

### Active Transport and Metabolizing Mechanisms

Finally, it is important to realize that in direct nose-to-brain transport, drug transporters and metabolizing capacity of nasal mucosa should be considered as well. Graff and Pollack (2003) have shown that P-glycoprotein may diminish brain accumulation of intranasally administered P-glycoprotein substrates (30). In addition, the metabolic capacity of the nasal mucosa can be modified by metabolizing enzymes (31). Wong and Zuo (2010) highlighted the importance and implications of how nasal metabolism might influence the transport of drugs via direct nose-to-brain transport. For instance, research has shown metabolizing enzymes (e.g., cytochrome P-450) are present in the nasal mucosa of rats and humans which might limit direct nose-to-brain transport of xenobiotics (32). Furthermore, qualitative and quantitative differences in nasal metabolic enzymes exist between animals and humans (33). Thus, taking into account only the differences in the relative absorption area of the nose in animals and humans alone might not be enough for successful translation of direct nose-to-brain transport from animals to humans.

## EVIDENCE FOR NOSE-TO-BRAIN DRUG AND DRUG DELIVERY SYSTEM TRANSPORT IN ANIMALS

Numerous animal studies have been performed in which IN delivery of drugs into the brain was investigated for various substances (e.g., small molecules, biologics, and specialized drug delivery systems). The main goal of this section is to highlight the large variety in the types of substances which were shown to enter the CNS via direct nose-to-brain transport (see Table II) without aiming to provide a complete list of all relevant substances, but merely to present a selection of studies where quantitative PK data was available that confirmed direct nose-to-brain drug transport. Kozlovskaya et al. (2014) have presented a more extensive list on the extent of direct nose-to-brain drug transport for a large amount of substances which were shown to enter the CNS via direct nose-to-brain transport (10). The extent was based on AUC values in brain and plasma following IV and IN drug administration, and expressed as drug targeting efficiency percentage (%DTE) and the nose-to-brain direct transport percentage (%DTP). Here, drug targeting efficiency percentage is calculated as

$$ \%\operatorname{DTE}=\left[\left({\operatorname{AUC}}_{\mathrm{brain}}/{\operatorname{AUC}}_{\mathrm{blood}}\right)\operatorname{in}\right]/\left[\left({\operatorname{AUC}}_{\mathrm{brain}}/{\operatorname{AUC}}_{\mathrm{blood}}\right)\mathrm{i}\mathrm{v}\right]\times 100\% $$

where AUC_brain_ is the area under the concentration-time curve of the compound in the brain (whole brain, or specific parts such as CSF), AUC_blood_ is the area under the curve of the compound in the systemic circulation (blood, or plasma, serum) and in and iv indicate intranasal or intravenous administration, respectively. The nose-to-brain direct transport percentage is calculated as

$$ \%DTP=\left(B{}_{\mathrm{in}}-B{}_{\mathrm{x}}\right)\kern0.28em /B{}_{\mathrm{in}}\times 100\% $$

With:

$$ B{}_{\mathrm{x}}=\left[B{}_{\mathrm{i}}{}_{\mathrm{v}}/P{}_{\mathrm{i}}{}_{\mathrm{v}}\kern0.28em \right].P{}_{\mathrm{i}}{}_{\mathrm{n}} $$

where *B*_x_ is the brain AUC fraction contributed by the systemic circulation through the BBB following IN administration, *B*_in_ is the brain AUC over time following IN administration, *P*_iv_ is the blood AUC over time following IV administration, and *P*_in_ is the blood AUC over time following IN administration.

**Table II:** Overview of Direct Nose-to-Brain Transport Animal Studies Cited in this Current Review

Type	Subtype	Author (year)	Substance(s)	Animal used
Small molecule drugs	N/A	Chou and Donnovan (1991) (29)	Procaine	Rats
			Tetracaine	Rats
			Bupivacaine	Rats
			Lidocaine	Rats
		Stevens *et al*. (2011) (34)	Remoxipride	Rats
		Stevens *et al*. (2009) (35)	Acetaminophen	Rats
		Westin *et al*. (2006) (36)	Morphine	Rats
Biologics	Peptides	Banks *et al*. (2004) (18)	Galanin-like peptide	Mice
		Nonaka *et al*. (2008) (22)	[Ser( (2))] exendin (1–9)	Mice
		Rat *et al*. (2011) (37)	Pituitary adenylate cyclase-activating polypeptide	Mice
		Gozes *et al*. (2000) (38)	Neuroprotective peptide NAP	Rats
		Sun *et al*. (2009) (39)	Calcitonin gene-related peptide	Rats
		Dhuria *et al*. (2009) (40)	Hypocretin-1	Rats
	Proteins	Thorne *et al*. (2004) (41)	Insulin growth factor 1	Rats
		Yu *et al*. (2005) (42)	Erythropoietin	Rats
		Ross *et al*. (2004) (43)	Interferon β1b	Rats
		Migliore *et al*. (2010) (44)	Ovalbumin	Rats
		Fliedner *et al*. (2006) (45)	Leptin	Rats
		Chen *et al*. (1998) (46)	Human nerve growth factor	Rats
		Yang *et al*. (2009) (47)	Vascular endothelial growth factor	Rats
		Ma *et al*. (2007) (48)	Human transforming growth factor β1	Rats
		Ma *et al*. (2008) (49)	Human basic fibroblast growth factor	Rats
	Other	Draghia *et al*. (1995) (50)	Adenoviral vector AdRSV βgal	Rats
		Han *et al*. (2007) (51)	pCMVβ and pN2/CMVβ	Mice
		Kim *et al*. (2009) (52)	αβ-Crystallin siRNA	Rats
		Danielyan (2009) (53)	Rat mesenchymal stem cells	Mice
Specialized drug delivery systems	N/A	Harmon *et al*. (2014) (54)	pGFP loaded nanoparticles	Rats
		Haque *et al*. (2014) (55)	Venlafaxine loaded alginate nanoparticles	Rats
		Fazil *et al*. (2012) (56)	Rivastigmine loaded chitosan nanoparticles	Rats
		Gao *et al*. (2007) (57)	Ulex europeus agglutinin I modified nanoparticles	Rats

This table summarizes a selection of animal studies which have evidence to confirm direct nose-to-brain transport of various drug categories (i.e., small molecule drugs, biologics, and specialized drug delivery systems). For a comprehensive overview, see Kozlovskaya *et al*. (2014) (10)

### Small Molecule Drugs

Several IN administered small molecule drugs have been shown to be delivered into the CNS via direct nose-to-brain transport. For example, Chou and Donnovan (1998) detected procaine, tetracaine, bupivacaine, and lidocaine in the CSF of the cisterna magna in rats following IN administration with a relative bioavailability (AUC_intranasal_/AUC_intra-arterial_) of 43% for procaine and 100% for tetracaine, buvicaine, and lidocaine (29). Procaine, tetracaine, bupivacaine, and lidocaine have distribution coefficients (LogD_octanol/water_) of −0.092, 2.18, 2.22, and 1.55. Therefore, the authors of this study argued that the low bioavailability of procaine might be caused by a lower distribution coefficient.

Furthermore, Stevens *et al*. (2011) showed that IN administered remoxipride had a total bioavailability of 89%. Total bioavailability was defined as the sum of the bioavailability to the central compartment (22%) and the bioavailability of direct nose-to-brain drug transport (67%) (34). This indicates that for remoxipride in the rat, the bioavailability to the brain by direct nose-to-brain transport is substantial. As lipophilic compounds such as remoxipride (LogP = 2.1) are generally more readily absorbed into the mucus layer than hydrophilic compounds (25,58), preferential absorption of remoxipride into the systemic circulation was expected to occur. However, this was not the case for remoxipride. This indicates that other processes seem to be involved that favor direct nose-to-brain transport, especially as the IN administration of the moderately lipophilic drug acetaminophen did not lead to brain distribution enhancement (35). Therefore, it seems that lipophilicity does not play an obvious role in direct nose-to-brain transport. Lastly, the opioid analgesic drug morphine was detected in brain homogenates of rats after IN administration (36). In comparison to IV drug administration a significantly faster distribution of morphine in the brain hemispheres was observed after initial transport through the olfactory bulb (36). Collectively, these results show several CNS-active small molecule drugs are transported into the CNS of rats via direct nose-to-brain transport.

### Biologics

Aside from small molecule drugs, peptides have also been shown to enter the CNS via direct nose-to-brain drug transport. Peptides are large molecules consisting of amino acid monomers which are covalently linked to each other with peptide bonds. Galanin-like peptide (MW = 6.5 kDa), [Ser( (2))] exendin (1–9) (MW = 980 Da), and pituitary adenylate cyclase-activating polypeptide (MW = 4.5 kDa) were observed in the CNS of mice after IN administration (18,21,22). Additionally, neuroprotective peptide NAP (MW 825 = Da), calcitonin gene-related peptide (MW = 3.8 kDa), and hypocretin-1 (MW = 3.6 kDa) were able to reach the CNS of rats by direct nose-to-brain transport mechanisms (37–40).

Proteins have also been shown to enter the CNS after IN drug administration in rats via direct nose-to-brain transport. Therefore, IN drug administration provides an interesting method to circumvent the BBB for proteins. Several studies have shown that the following proteins were present in the CNS of rats after IN administration: insulin growth factor 1 (MW = 7.65 kDa), erythropoietin (30–34 kDa), interferon β1b (MW = 18.5 kDa), ovalbumin (45 kDa), leptin (16 kDa), human nerve growth factor (MW = 26.5 kDa), vascular endothelial growth factor (MW = 38.2 kDa), human transforming growth factor β1 (MW = 25 kDa), and human basic fibroblast growth factor (MW = 17.2 kDa) (41–49). All these studies have evidence that initial transport into the brain occurs by direct nose-to-brain transport via the olfactory and trigeminal route.

In addition, few studies have focused on direct nose-to-brain delivery of gene vectors and stem cells. The ability to modify gene expression in CNS cells could be a promising technique to treat chronic CNS diseases. For instance, a study performed by Draghia *et al*. (1995) showed rats expressed β-galactosidase in the brain after IN administration of replication-defective adenoviral vector AdRSV β gal (50). The affected structures in the brain were the olfactory bulb, olfactory nucleus, locus coeruleus, and area postrema. Furthermore, direct nose-to-brain transport has also been observed in mice which were IN administered with pCMVβ and pN2/CMVβ (51). Also, small interfering RNA (siRNA) to αβ-crystallin was successfully delivered into the CNS of rats after IN administration which resulted in a gene knockdown in the olfactory bulb, amygdala, and hypothalamus (52). Lastly, fluorescently labeled rat mesenchymal stem cells (MSC) have been observed in the olfactory bulb, hippocampus, thalamus, cortex, and subarachnoid space of mice 1 hour after direct nose-to-brain transport (53). IN administration of stem cells might be an interesting technique to treat neurodegenerative diseases.

### Specialized Drug Delivery Systems

Over the past few years, specialized drug delivery systems have been more extensively studied. For example, Harmon *et al*. (2014) observed successful transfection of rat brain cells adjacent to capillary endothelial cells located in the rostral-caudal axis of the brain. This effect was produced by giving rats an IN administration of unimolecularly compacted nanoparticles which contained plasmid DNA encoding green fluorescent protein (54). Although exact transport mechanisms were not studied, green fluorescent protein expression was shown to be significantly higher after using encapsulated plasmid DNA than after using naked plasmid DNA.

Furthermore, direct nose-to-brain transport of venlafaxine loaded alginate nanoparticles has been shown in rats (55). Venlafaxine transport was significantly higher upon encapsulation into alginate nanoparticles and subsequent IN administration. Moreover, confocal laser scanning microscopy results show rhodamin loaded alginate nanoparticles enter the brain intact. Lastly, IN administration of rivastigmine loaded chitosan nanoparticles in rats resulted in higher rivastigmine brain concentrations than IV administration, as determined by the brain homogenate method (56). Fazil *et al*. (2012) used confocal laser scanning microscopy to qualitatively assess the biodistribution of rhodamin loaded chitosan nanoparticles in the brain, which indicated chitosan nanoparticles were intact upon entering the brain.

Aside from nanoparticles, many other techniques are being developed which could enhance direct nose-to-brain drug transport. For instance, using chitosan (coated) nanoparticles or cyclodextrin inclusion complexes provides a way to improve the solubility of poorly soluble drugs (13,59). Transport of poorly soluble drugs can also be enhanced by using bio-adhesive emulsions, which have been demonstrated to increase the brain uptake of small molecule drugs (13). An interesting approach to reduce clearance and to achieve targeted delivery involves the surface modification of nanoparticles to contain ligands which bind to specific cell types. For example, nanoparticles covered with ulex europeus agglutinin I, a ligand which binds to receptors present in the olfactory region, have been shown to result in a higher drug transport than unmodified nanoparticles when administered IN in rats (57).

## EVIDENCE FOR NOSE-TO-BRAIN DRUG TRANSPORT IN HUMANS

Although direct nose-to-brain drug transport has obtained increased attention, only one study so far has collected quantitative PK data which confirmed this type of transport in humans (60). Born *et al*. (2002) obtained PK evidence of direct nose-to-brain transport in humans after IN administration of the peptides melanocortin (4–10), vasopressin, and insulin (60). Peptide concentrations were measured in CSF and systemic blood. CSF samples were taken from the interspace between the fourth and fifth lumbar vertebrae. After IN administration, melanocortin (4–10) and insulin concentrations in the CSF increased while plasma levels remained stable in comparison to baseline levels. However, IN administered vasopressin was also present in systemic blood, suggesting that either transport across the BBB and/or direct uptake in the systemic circulation has been involved. In contrast, Merkus *et al*. (2003) was unable to confirm direct nose-to-brain transport of melatonin and hydroxocobalamin, on the basis of plasma and CSF concentrations obtained in humans, when comparing the extent of drug transport enhancement after IN and IV administration (61). This might be caused by preferential absorption of melatonin and hydroxocobalamin into the systemic circulation (61). In conclusion, direct nose-to-brain drug transport in humans remains an unsolved issue.

Direct nose-to-brain drug transport in humans can also be assessed indirectly, via a non-quantitative approach by measuring drug-specific PD. In many studies, IN administration of the peptide oxytocin has been investigated (62). As summarized by Chapman *et al*. (2013), IN administration of oxytocin has been shown to increase trust in humans. These results indicate transport through direct nose-to-brain transport mechanisms as research performed by McGonigle (2012) has demonstrated that oxytocin is unable to cross the BBB in significant quantities (63). Thus, effects are likely to be caused by oxytocin which entered the brain through direct nose-to-brain mechanisms. However, no information was obtained about the PK such as extent of drug transport into the brain.

Collecting quantitative PK evidence for direct nose-to-brain transport in humans is difficult for several reasons. First of all, many ethical issues arise when performing studies in humans. Also, researchers have to perform human studies according to laws and regulations developed to protect human subjects from potential misconduct, which causes such studies in humans to require more time and money than studies in animals. Also, there are practical considerations. Ideally, researchers need to monitor unbound drug concentrations at the CNS target site in humans (3). However, due to the ethical inaccessibility of the human brain for sampling, this is not possible in humans. Therefore, CSF is usually chosen as a surrogate for brain ECF concentrations due to its accessibility via the spinal cord. However, drug concentrations in CSF might not fully reflect drug concentrations found in brain ECF (64–66). Furthermore, the collection of CSF samples in humans requires highly specialized medical personnel. In conclusion, performing quantitative nose-to-brain studies in humans is complex and expensive but in selected cases validations are essential to obtain an improved understanding of human nose-to-brain drug delivery.

## TRANSLATIONAL PK-PD OF NOSE-TO-BRAIN REMOXIPRIDE TRANSPORT IN RATS

Translational investigations of direct nose-to-brain transport from rats to humans could be of great significance for the development of IN delivered CNS-active drugs. However, an improved understanding of the predictive value of rats as a preclinical animal model requires a mechanistic and structured approach. First we should learn from preclinical translation between different conditions, especially PK-PD following IV and IN drug administration. This is exemplified by the following, so far unique, example. The PK model developed by Stevens *et al*. (2011) shows the added value of separation and quantitation of systemic and direct nose-to-brain transport after IN administered remoxipride in freely moving rats, in terms of *extent and rates* (34). As anesthesia and stress significantly influence nose physiology, an advanced rat model for IN administration in freely moving animals under minimum stress conditions was earlier developed and used accordingly (35). Remoxipride was administered at different dosages, in freely moving rats, by the IN and IV route. The plasma and brain ECF data were simultaneously analyzed using non-linear mixed effects modeling to identify the existence of direct nose-to-brain transport in a quantitative manner. This PK model was then linked to a PD model that could successfully predict PD also after IN administration, indicating the validity of the structural PK-PD model.

### PK Modeling of IV and IN Administered Remoxipride in Rats

**Fig. 2:**
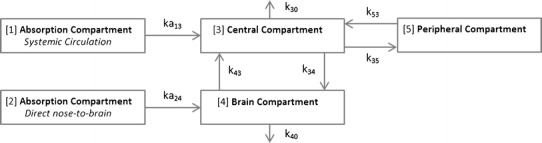
PK model of remoxipride delivered through IN administration. Compartment numbers are shown between *brackets*. Two absorption compartments exist, which describe absorption of drug into the central compartment (*systemic circulation*) and another one which describes direct drug absorption into the brain (*direct nose-to-brain transport*). The central compartment represents the remoxipride concentrations in plasma. Distribution of drug into other tissues and organs is described by the peripheral compartment. Lastly, the brain compartment represents drug concentrations in the ECF (34). Parameter values are provided in Table III

In Fig. 2, a novel PK model of remoxipride in rats after IN administration is shown (34). This multi-compartment PK model was developed by concurrent modeling of unbound remoxipride concentrations in plasma and brain ECF, which were collected following IV as well as IN administration (see Table III for parameter description and values). Two absorption compartments were identified in this model to describe (1) the absorption rate constant of remoxipride from the nasal cavity into the central compartment (ka_13_) and (2) the absorption rate constant via direct nose-to-brain transport (ka_24_). Remoxipride concentrations in plasma were represented by the central compartment and the elimination rate constant of remoxipride from plasma was included (k_30_). Furthermore, a peripheral compartment was incorporated to account for remoxipride distribution rate constants from plasma into peripheral tissues and organs (k_35_ and k_53_). Concentrations of remoxipride in brain ECF were described by the brain compartment, with brain elimination, and transport rate constants of remoxipride across the BBB (k_34_ and k_43_ for inward and outward transport, respectively). This model was subsequently evaluated by performing a visual predictive check (VPC), showing that the simulated data by the model were comparable to actual data obtained in the rat studies, both following IV and IN remoxipride administration indicating the validity of the structural PK model.

**Table III:** Parameter Estimates of Remoxipride PK in Rats Following IN Administration

Estimated parameters	Parameter description	Value	CV%
ka_13_ (/h)	Absorption rate constant into the central compartment (systemic circulation)	1.54	11.8
ka_24_ (/h)	Absorption rate constant of direct nose-to-brain transport	0.033	43.8
k_30_ (/h)	Elimination rate constant from the central compartment	1.12	10.1
Fk_40_ (/h)	Elimination rate constant from the brain compartment as fraction of k_30_	0.302	17.5
V3 (l/kg)	Volume of distribution (central compartment)	0.088	13.6
V4 (l/kg)	Volume of distribution (brain compartment)	0.873	24.1
V5 (l/kg)	Volume of distribution (peripheral compartment)	0.417	8.60
Q4 (l/h/kg)	Intercompartmental clearance between the central compartment and the brain compartment	0.70	19.9
Q5 (l/h/kg)	Intercompartmental clearance between the central compartment and the peripheral compartment	1.20	10.2
F_TOT_	Total bioavailability	0.89	4.60
F_1_	Bioavailability attributable to transport via initial absorption into the central compartment and subsequent transport across the BBB	0.22	20.1
F_2_	Bioavailability attributable to transport directly from the nose into the brain	0.67	N/A
RV plasma	Residual value of plasma	0.098	N/A

Estimated parameters are shown for the compartments presented in Fig. 2

*CV* coefficient of variation, *F* bioavailability, *k* elimination rate constant, *ka* absorption rate constant, *Q* inter-compartmental clearance, *V* volume of distribution, *RV* residual variability (34)

For absorption, in terms of extent, the total (absolute) bioavailability of remoxipride following IN administration was 89%. This was the sum of nose-to-systemic absorption (F_1_, 22%) and direct nose-to-brain absorption (F_2_, 67%). This indicates that direct nose-to-brain transport of remoxipride was more extensive than nose-to-systemic absorption. In terms of rates, remoxipride absorption from nose-to-systemic compartment (ka_13_) was faster than absorption from nose-to-brain (ka_14_). Interestingly, remoxipride elimination from brain ECF (ka_40_) was shown to be significantly smaller after IN drug administration than after IV drug administration. This suggests the existence of flip-flop kinetics (absorption-rate limited elimination of remoxipride from brain ECF).

Brain distribution, in terms of extent, was restricted as indicated by the 0–4 h AUC (brain ECF)/AUC (plasma, unbound) ratios (K_puu_) values of 0.3 and 0.2 after IN and IV administration, respectively (which is an underestimation for the IN administration, as unanticipated, the elimination half-life was absorption-rate limited such that AUC 0–∞ values could not be adequately determined). Then, in terms of rates, transport across the BBB (k_34_ for the rate constant into and k_43_ for the rate constant out of the brain) was determined to be linear, indicating remoxipride transport mainly relied on passive diffusion. Nonetheless, linearity in transport across the BBB should never be automatically assumed. For instance, influx and efflux transporters might both influence the distribution of drugs between the central compartment and the brain compartment and lead to a net impression of linear transport in a certain concentration range.

In conclusion, this study demonstrated successful separation and quantitation of systemic and direct nose-to-brain transport in rats after IN administration of remoxipride, in terms of extent as well as rate. The information on absorption rates is important for the resulting PK profiles in the brain that drive CNS drug effects, which is ultimately what we are interested in.

### PK-PD Modeling of IV and Translation to IN Administered Remoxipride in Rats

**Fig. 3:**
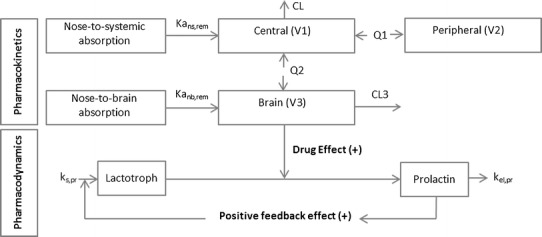
Mechanism-based PK-PD model of remoxipride delivered in rats via IN drug administration. The PK part was based on the compartment model of Stevens *et al*. (2011) (34) and the PD part on a pool model developed by Movin-Osswald *et al*. (1995) (67)

The translational PK model as described above was further developed into a translational PK-PD model using pituitary hormone prolactin plasma levels as PD readout (67,68) (Fig. 3). Prolactin is synthesized in the lactotrophs of the pituitary gland and its release into plasma will occur upon dopaminergic inhibition. Remoxipride, a dopaminergic D2 receptor antagonist, is known to elevate plasma prolactin levels following release from the lactotrophs in the pituitary gland. After assessment of baseline variation in prolactin plasma concentrations, the prolactin plasma concentrations were measured upon single IV doses of remoxipride, as well as following double low IV dosing of remoxipride with different time intervals to get information on the rate of synthesis of prolactin in the lactotrophs of the pituitary gland (67). The final PK-PD model comprised the PK model for remoxipride concentrations in plasma and brain ECF; a dopamine antagonism component for the effect of remoxipride brain ECF concentrations on stimulation of prolactin release; a pool model incorporating prolactin synthesis and storage in lactotrophs, as well as release into and elimination from plasma; and a homeostatic feedback component being the positive feedback for prolactin plasma concentrations on prolactin synthesis in the lactotrophs (34).

Subsequently, the translational value of this PK-PD relationship developed for IN administration was evaluated on its translational value with regard to IN administration. Thus, the dataset obtained in rats following IN administration of the different doses of remoxipride was used. The PK-PD relationship of remoxipride following IN administration could be adequately predicted by simulations of the PK-PD model, demonstrating successful translation of remoxipride PK in rats between the two distinct routes of drug administration.

## TRANSLATIONAL PK AND PK-PD OF IV REMOXIPRIDE FROM RATS TO HUMANS

Successful mathematical modeling of unbound remoxipride PK in plasma and brain ECF in rats after IV and IN drug administration, as shown in the previous study example (34), provided the basis for the “humanized” PK and finally the PK-PD model (68). For translation of the preclinical PK model to humans, allometric scaling was applied and the “humanized” PK model successfully predicted existing plasma PK of remoxipride as measured in humans (67). As no information on human brain ECF concentrations were available, BBB transport scaling from rat to human was made under the assumption of being linear as was the case in rats. Thus, brain ECF PK in humans was predicted, without the possibility of validation on existing human data.

However, in rats, the brain ECF PK was found to be indistinguishable from target site concentrations (i.e., showing a direct and reversible effect on prolactine release from the pituitary gland). Also, in the study by Movin-Osswald and Hammarlund-Udenaes *et al.* (1995)\Phuman plasma prolactin concentrations were assessed. Therefore, this PD information could be used to see if the PD obtained in humans would be adequately predicted by the “humanized” PK-PD model for which drug-specific and biological-system-specific parameters were obtained from the literature. Indeed, it was demonstrated that the humanized PK-PD model could satisfactorily predict PK-PD relationship of remoxipride in humans. This indicates that the addition of PD measurements can be of added value in the development of preclinically derived PK-PD models for CNS active drugs.

## TOWARDS PREDICTION OF NOSE-TO-BRAIN TRANSPORT IN HUMANS

**Fig. 4:**
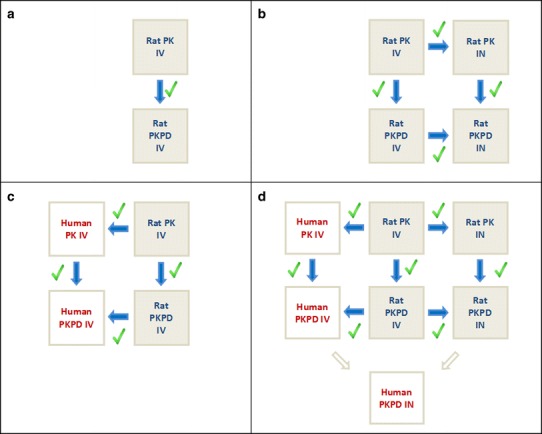
Global overview of steps in development of translational PK-PD models, as exemplified for remoxipride (34,67). First, a rat PK model is developed on data following IV administration only, followed by inclusion of also PD data, and simulated data by the model are validated on observed data (**a**). Then, these models are further developed to include IN administration in the rat, and simulated data are validated on their prediction of obtained PK and PK-PD data following IN administration (**b**). Subsequently, the rat IV PK and PK-PD models are “humanized” by appropriated scaling and simulated data by the humanized model is validated on obtained human PK and PK-PD data (**c**). Finally, it would be possible to further develop the humanized PK and PK-PD model as developed for IV administration, by further scaling of nose characteristics from rat to human

Translational PK and PK-PD models developed on the basis of preclinical data on CNS active drugs is a promising approach to improve prediction of CNS target site concentrations in human and associated effects. For drugs that have difficulties in distribution to the brain, the IN route of drug administration may be a good alternative as a direct absorption from the nose to the brain has been proven to exist. The question is now how we can progress in predicting nose-to-brain transport in humans. Here, we provide considerations for translational preclinical studies, first those following IV administration as a basis, then extended with special considerations following IN administration (see Fig. 4 for the flow in model development).

For the translation of animal to human PK and PK-PD following IV administered drugs on the basis of preclinical data, preclinical experiments will substantially improve if we include:

- *unbound drug concentrations*, as it is the unbound drug concentration that drives transport processes (BBB transport, intra-brain distribution, unbound brain concentrations) and target interactions that lead to drug effects (69,70). Brain ECF data are often highly appropriate as many targets are facing the ECF. However, also intracellular unbound concentrations may be needed (69–71). If CSF concentrations are used, then the relationship between brain ECF and CSF should be considered (64–66,72).
- *longitudinal measurements* (PK, PD, disease stage), as hereby information on rate and extent of mechanisms can be unraveled (73).
- *combined measurements* on different levels of biomarkers (plasma PK, brain PK, target interaction, brain PD, signal transduction, disease (74)), in single subjects, as data are connected (inter-dependency of processes) and context (site of administration, species, etc.) dependent, as knowledge on only one individual processes is worthless and its role should be investigated in multiple contexts. Also, to have information on what concentrations can actually represent target site concentrations, measurement of concomitant effects of the drug is needed.
- *influence of drug properties*, as the combination of drug properties and biological system characteristics will determine the PK, and PK-PD
- *advanced mathematical modeling* to integrate all data for the development of preclinical and “humanized” mathematical models, including statistical approaches to obtain insight into sources of variability. Advanced mathematical modeling techniques are needed to reveal complex relationships of body processes and interactions of the body and the drug to be ultimately integrated into mathematical models.
- *human data* to test validity of predictions of the “humanized” models.

The translation of drug PK and PK-PD from animals to humans following IN administration will obviously be more complicated than the examples presented above. This could make translational investigations of IN administered drugs more difficult but not impossible. To further work on the prediction of human PK and PK-PD following IN administration, in addition to the aforementioned points, we need to consider the:

- *explicit distinction between the different absorption*/*transport routes* (i.e., absorption directly from nose to the brain, absorption from the nose into the systemic circulation, and distribution between the systemic circulation and the brain), as the rate and extent of each absorption/transport pathway may independently differ between rats and humans.
- *inclusion of nose characteristics* that differ between preclinical species and humans.
- *nasal metabolism and active transport* as this may implicate correct measurements due to the influence of other processes.
- *influence of formulations* as absorption may be influenced by formulation.

## CONCLUSIONS

Over the past few decades, numerous IN administered substances, such as small molecules, biologics, and specialized drug delivery systems, have been shown to enter the CNS of animals via direct nose-to-brain transport while bypassing the BBB. Circumvention of the BBB is facilitated by extracellular and intracellular transport processes of drugs along the olfactory nerve and the trigeminal nerve which provide direct entry points to the CNS. However, direct nose-to-brain transport is mostly dependent on transport along the olfactory nerve.

IN drug administration is non-invasive which means it could be a promising drug delivery method for patients who suffer from (chronic) CNS diseases. Preclinical animal studies show encouraging results, confirming direct nose-to-brain transport. However, multiple anatomical, physiological, and histological differences exist between animals and humans. Therefore, it is of great importance to investigate the predictive value of preclinical animal models within the context of direct nose-to-brain transport. This can be investigated by using an integrated approach which is based on the collection of quantitative PK and PK-PD data over time.

Quantitative PK and PK-PD data on unbound drug concentrations after IN *versus* IV administration obtained from animals provides the opportunity to create advanced mathematical PK and PK-PD models that can be scaled to humans. Using such an integrated approach would provide insight into the predictive value of preclinical animal models. This would benefit the development of intranasally administered CNS-active drugs.

## Abbreviations

%DTE: Drug targeting efficiency percentage
%DTP: Nose-to-brain direct transport percentage
BBB: Blood–brain barrier
BCSFB: Blood cerebrospinal fluid barrier
CNS: Central nervous system
CSF: Cerebrospinal fluid
ECF: Extracellular fluid
logD: Distribution coefficient
logP: Partition coefficient
IN: Intranasal
IV: Intravenous
K_puu_: The extent of tissue distribution (ratio of AUC or clearance values) based on unbound concentrations in plasma and the tissue
MRPs: Multidrug resistance-related proteins
MSC: Mesenchymal stem cells
MW: Molecular weight
NCSA: Nasal cavity surface area
NCV: Nasal cavity volume
OSNs: Olfactory sensory neurons
Pgp: P-glycoprotein
PK: Pharmacokinetic(s)
PD: Pharmacodynamic(s)
PK-PD: Pharmacokinetic–pharmacodynamic
siRNA: Small interfering RNA
VPC: Visual predictive check
